# RIGD: A Database for Intronless Genes in the Rosaceae

**DOI:** 10.3389/fgene.2020.00868

**Published:** 2020-08-07

**Authors:** Tianzhe Chen, Dandan Meng, Xin Liu, Xi Cheng, Han Wang, Qing Jin, Xiaoyu Xu, Yunpeng Cao, Yongping Cai

**Affiliations:** ^1^School of Life Sciences, Anhui Agricultural University, Hefei, China; ^2^Anhui Provincial Engineering Technology Research Center for Development & Utilization of Regional Characteristic Plants, Anhui Agricultural University, Hefei, China; ^3^Key Laboratory of Cultivation and Protection for Non-Wood Forest Trees, Ministry of Education, Central South University of Forestry and Technology, Changsha, China

**Keywords:** intronless genes, gene annotations, platform, database, Rosaceae

## Abstract

Most eukaryotic genes are interrupted by one or more introns, and only prokaryotic genomes are composed of mainly single-exon genes without introns. Due to the absence of introns, intronless genes in eukaryotes have become important materials for comparative genomics and evolutionary biology. There is currently no cohesive database that collects intronless genes in plants into a single database, although many databases on exons and introns exist. In this study, we constructed the Rosaceae Intronless Genes Database (RIGD), a user-friendly web interface to explore and collect information on intronless genes from different plants. Six Rosaceae species, *Pyrus bretschneideri, Pyrus communis*, *Malus domestica*, *Prunus persica*, *Prunus mume*, and *Fragaria vesca*, are included in the current release of the RIGD. Sequence data and gene annotation were collected from different databases and integrated. The main purpose of this study is to provide gene sequence data. In addition, attribute analysis, functional annotations, subcellular localization prediction, and GO analysis are reported. The RIGD allows users to browse, search, and download data with ease. Blast and comparative analyses are also provided through this online database, which is available at http://www.rigdb.cn/.

## Background

Genes in eukaryotes are generally composed of exons and introns, and according to the presence and absence of introns, they can be divided into intron-containing genes and intronless genes. It is generally believed that intron number is closely related to the complexity of the eukaryotic genome. If an organism is complex, it has more introns (Sakharkar et al., 2004). Most eukaryotic genes have two or more introns, while prokaryotes have a large number of intronless genes (Rogozin et al., 2005). Intronless genes are not interspaced by introns and can be sequentially encoded into proteins. Intronless genes can serve as focal point in analyses of gene function and evolution. For example, compared with intron-containing homologs, intronless genes can be used as a model to study the important role of introns, which are only found in eukaryotes (Tine et al., 2011). Furthermore, studies on intronless genes help to solve some evolutionary issues, including (1) the main factors leading to the emergence of intronless genes (gene duplication, inheritance from ancient prokaryotes, retroposition or other mechanisms), (2) the evolutionary significance of retroposition (retrogenes are considered to be intronless), and (3) the biological origins of introns (is the introns-early hypothesis or introns-late hypothesis more correct) (Sakharkar and Kangueane, 2004).

In eukaryotes, the proportion of intronless genes varies from 2.7 to 97.7% of the genome (Louhichi et al., 2011). Currently, researchers have identified intronless genes in some species of mammals, hindmouths, bony fish, and plants (Agarwal and Gupta, 2005; Sakharkar et al., 2006; Jain et al., 2008; Zou et al., 2011). In Jain et al. (2006) studied the early auxin response SAUR (small auxin-up RNA) gene family in rice and found that all 58 members of the gene family were intronless genes. In the process of studying the functions of gene families in *Arabidopsis*, researchers also found a large number of intronless genes in the f-box protein family, DEAD box RNA helicase family, and PPR (pentatricopeptide repeat) gene family (Aubourg et al., 1999; Lecharny et al., 2003; Lurin et al., 2004). In addition, some of the largest families, such as the G-protein receptor family and the olfactory receptor family, are also composed of intronless genes (Gentles and Karlin, 1999; Takeda et al., 2002). Currently, the most studied intronless gene is the histone gene in the human genome. Researchers aim to explore the role of intronless genes in life processes by studying these gene families.

Since researching intronless genes in eukaryotes can help researchers better understand the evolutionary mechanism of related genes and genomes, the study of intronless genes has attracted more and more attention. In recent years, the construction of intronless gene databases has attracted great attention as the research on intronless genes. Relevant databases can provide important data resources for functional and evolutionary studies, facilitate researchers to carry out relevant research. So far, there are mainly databases on intronless genes: GENOME SEGE (Sakharkar and Kangueane, 2004), IGD (Louhichi et al., 2011), PIGD (Yan et al., 2014), and IGDD (Yan et al., 2016). GENOME SEGE contains NCBI data regarding the intronless genes of eukaryotes, however, the database website has stopped updating the data, and users are unable to access it. The IGD database, which includes 687 human intronless genes, was published in 2011. PIGD provides a platform for the collection, integration, and analysis of intronless genes in Poaceae. IGDD provides a comprehensive platform for researchers to explore intronless genes in dicot plants.

To build a centralized platform, we present the Rosaceae Intronless Genes Database (RIGD)^1^. This database, with a user-friendly web interface, covers a collection of intronless genes from six genome-sequenced Rosaceae species. The RIGD integrates functional and evolutionary annotations, making it easy for researchers to find content of interest and download detailed information. The RIGD provides a comparative analysis of genome data from six species in conjunction with the Blast program. Compared to the databases specifically for individual organisms, we expect the RIGD to be a useful resource for the research community, especially for studies on molecular function and the evolution of intronless genes.

## Construction and Content

### Data Sources

Currently, the RIGD includes the following six Rosaceae species: *Pyrus bretschneideri*, *Pyrus communis*, *Malus domestica*, *Prunus persica*, *Prunus mume*, and *Fragaria vesca*. Genome data of *Malus domestica* and *Prunus mume* were downloaded via FTP from the NCBI genomes database^2^. Genome data of the other species were downloaded from the GDR database (Jung et al., 2019)^3^ (Table 1).

**TABLE 1:** The sources of six species in RIGD.

**Species**	**Sources**
*Pyrus bretschneideri*	GDR (ftp://ftp.bioinfo.wsu.edu/www.rosaceae.org/Pyrus_x_bretschneideri/Pbretschneideri-genome.v1.1)
*Pyrus communis*	GDR (ftp://ftp.bioinfo.wsu.edu/species/Pyrus_communis/Pcommunis_DH_genome.v2.0)
*Malus domestica*	NCBI (ftp://ftp.ncbi.nlm.nih.gov/genomes/all/GCF/002/114/115/GCF_002114115.1_ASM211411v1)
*Prunus persica*	GDR (ftp://ftp.bioinfo.wsu.edu/species/Prunus_persica/Prunus_persica-genome.v2.0.a1)
*Prunus mume*	NCBI (ftp://ftp.ncbi.nlm.nih.gov/genomes/all/GCF/000/346/735/GCF_000346735.1_P.mume_V1.0)
*Fragaria vesca*	GDR (ftp://ftp.bioinfo.wsu.edu/species/Fragaria_vesca/Fvesca-genome.v4.0.a1)

### Identification of Rosaceae Intronless Genes

A set of strict standards was used to identify Rosaceae intronless genes. First, we used a Perl script to extract genes containing only one line of “exon” from each genome information in the genome annotation files (GFF/GFF3 format files) and then used them as candidate intronless genes for further screening. The basis for the screening was: if there was only one row of “exons” in the genome information, indicating that the coding sequence is not disrupted by an intron, then the gene is an intronless gene. Since the mitochondrial genes and chloroplast DNA do not contain introns, the genes annotated as “Mt” (mitochondria) and “Pt” (chloroplast) were rejected. In addition, genes that are not mapped to the chromosome were removed. Genes defined as “pseudogene” or “transposable element” in the annotation files were deleted because a pseudogene cannot be transcribed or translated, it is usually not functional. Through the above steps, we obtained no redundant intronless genes in six Rosaceae species. Using the identified intronless gene number, we used a Perl script to extract the protein sequence and CDS sequence of the intronless genes and renumber them according to certain criteria.

### Intronless Gene Annotation

**FIGURE 1:**
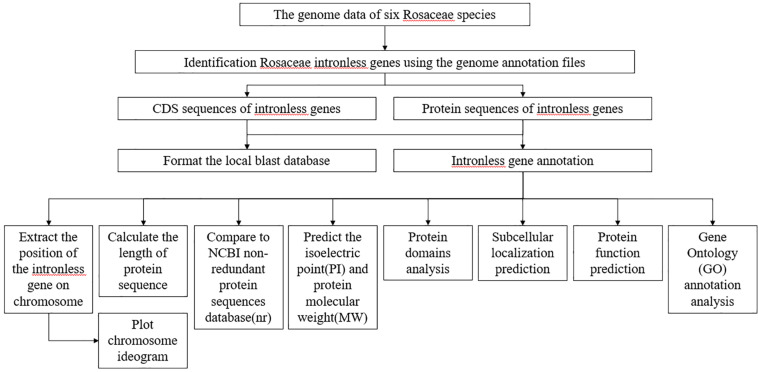
The flowchart describing the analysis of RIGD. After the identification of intronless genes, attribute analysis, functional annotations, subcellular localization prediction, and GO analysis were accomplished.

We established the following procedure to analyze each intronless gene stored in RIGD: (Figure 1). (1) A Perl script was used to extract the position information for intronless genes on corresponding chromosomes, and calculate the length of protein sequences. (2) Chromosome ideograms were plotted by using the chromosomeplot tool in MATLAB software^4^ (Snijders et al., 2001). (3) The protein sequences were compared to the NCBI non-redundant protein sequences database (nr)^5^ by using Diamond with default parameters^6^ (Buchfink et al., 2015). The GI numbers were obtained, and then the bioperl module^7^ was used to submit GI numbers to NCBI for the corresponding annotation information. (4) A Python script was used to submit the protein sequences to ExPASy^8^ (Artimo et al., 2012) to predict the isoelectric point (pI) and protein molecular weight (Mw). (5) Subcellular localization was predicted with MultiLoc2^9^ (Blum et al., 2009). (The parameters are set as follows: -origin = plant, -predictor = HighRes, -output = advanced) (6) Protein domains were analyzed by using InterProScan (Jones et al., 2014) with default parameters and searched the Pfam database (El-Gebali et al., 2019). (7) Protein function was predicted based on sequences and structural templates from DeepGOPlus (Kulmanov and Hoehndorf, 2019), I-TASSER (Iterative Threading ASSEmbly Refinement) (Roy et al., 2010; Yang and Zhang, 2015; Yang et al., 2015) and InterProScan with default parameters. Finally, (8) eggNOG mapper (Huerta-Cepas et al., 2017, 2019) was used to implement Gene Ontology (GO) annotation analysis. (The parameters are set as follows: -m diamond –tax_scope auto –go_evidence non-electronic –target_orthologs all –seed_ortholog_evalue 0.001 –seed_ortholog_score 60 –query-cover 20 –subject-cover 0). Visualization of GO categories was performed by using the WEGO online tool (Ye et al., 2006, 2018).

### Comparisons Between Rosaceae Species

In addition to analyzing the intronless genes of six Rosaceae species, we conducted the following comparative analysis of the intronless genes among species: (1) the number and percentage of intronless genes in each chromosome, (2) the distribution of protein length, (3) the distribution of pI, (4) the distribution of Mw, (5) the statistics of subcellular localization, and (6) the statistics of GO classifications.

### The RIGD Implementation and Web Interface

**FIGURE 2:**
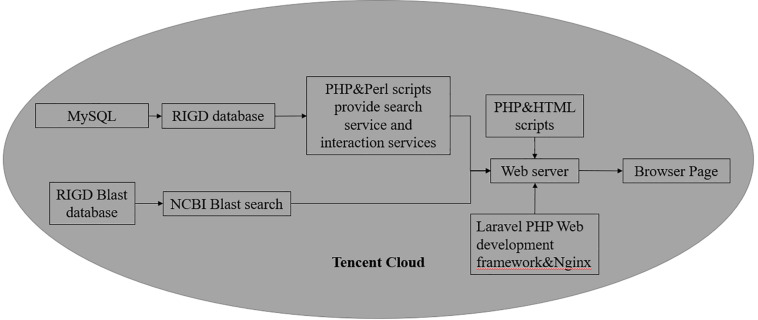
The Flowchart of RIGD Sitemap. All the data is stored in MySQL database, the control of the platform is implemented by PHP and Perl scripts.

As a web-based platform, the RIGD is constructed in a Tencent cloud server, and the operating system is Ubuntu Server 16.04.1 LTS 64-bit. The RIGD combines the MySQL (version 8.0.17) database management system with a dynamic web interface based on PHP (version 7.3.9-1), Laravel (version 5.8), Nginx (version 1.10.3), and Perl (version 5.22.1) scripts (Figure 2).

## Utility and Discussion

### Web Interface

**FIGURE 3:**
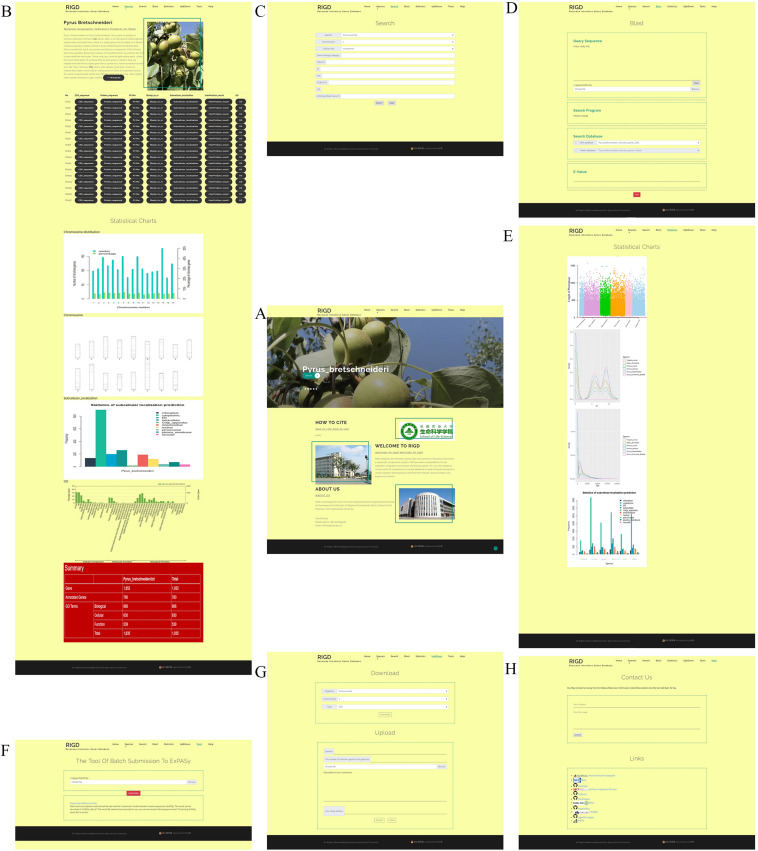
An overview of the website page in the RIGD. **(A)** Home interface show photographs of six species, the RIGD’s project description, author information and contact information. **(B)** In Species interface, the description of species, the download links of relevant analysis data and the analysis results are presented. **(C)** In Search interface, researchers can use different types of keywords to obtain the required data. **(D)** Blast interface can be used for Blast comparison of intronless genes in RIGD. **(E)** Statistics interface show the result of comparative analysis between Rosaceae species. **(F)** Tools interface support a tool that can batch submit sequences to ExPASy for pI/Mw prediction. **(G)** Upload&Download interface is designed to download and upload data. **(H)** Contact us interface. Researchers can contact us by email and link to some tools we used in our work.

The web interface of the RIGD is designed to comprise the following seven components: Home, Species, Search, Blast, Statistics, Upload&Download, and Contact Us. The RIGD provides a user-friendly interaction experience (Figure 3).

### Home

The RIGD has seven navigation bars at the top. Scrolling through the home page reveals large photos of six species of Rosaceae. There is a “detail” button on each photo that can be clicked to link to the “species” interface for each species. In addition, the RIGD’s project description, author information and contact information are also available on the home page.

### Species

**FIGURE 4:**
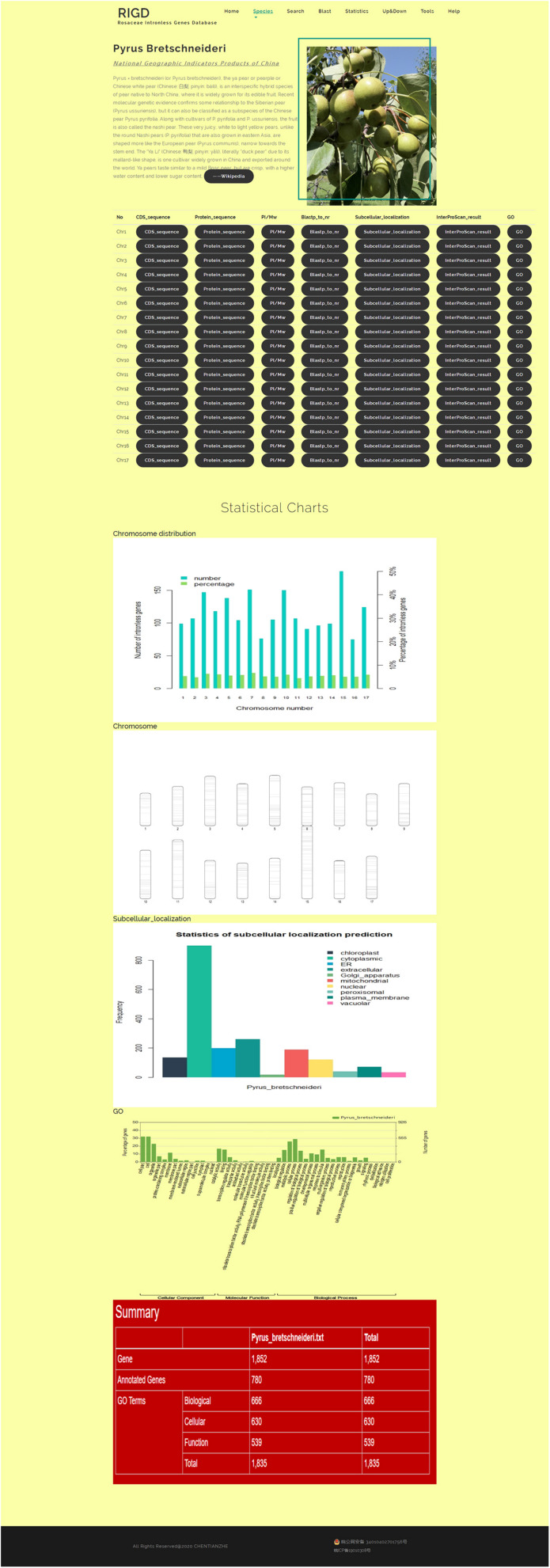
The website page of Species. In addition to the download links to the data, five statistical charts show the results of the number and proportion of intronless genes on each chromosome, the statistics of subcellular localization prediction, the chromosome ideogram, and the statistics of GO classifications.

The bar opens a drop-down menu with the names of the six Rosaceae species covered in the RIGD. Clicking to enter, you can then see a detailed description of the species and the picture on the page. There is also a table with download links, where much of data is available, including the CDS and protein sequence of intronless genes, the prediction of isoelectric point and protein molecular weight, the results of the sequence compared with the nr database, the results of protein domain analysis, the results of subcellular localization prediction, the results of protein function prediction, and the results of GO analysis. Some statistical charts are also shown on the page, such as the number and proportion of intronless genes on each chromosome, the statistics of subcellular localization prediction, the distribution of pI and Mw, and the statistics of GO classifications (Figure 4).

### Search

**FIGURE 5:**
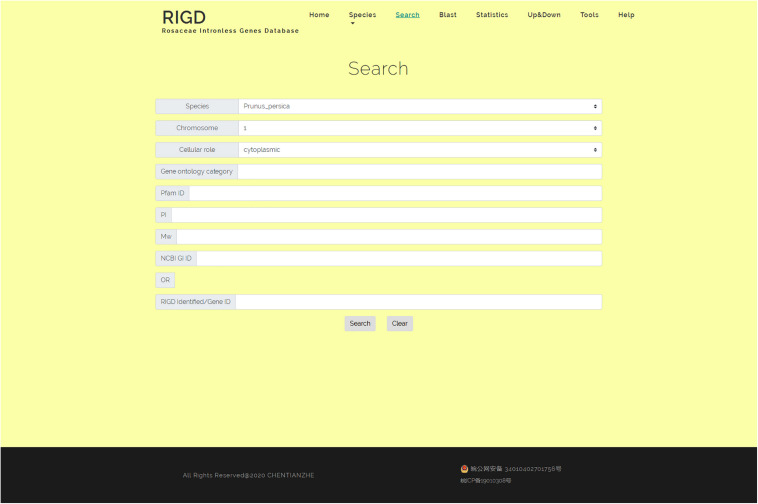
The website page of Search. Researchers can search the RIGD database by species name, chromosome number, classification of subcellular location prediction, GO number, Pfam ID, the value of pI or Mw and NCBI GI ID.

In the search interface, users can search by species name, chromosome number, classification of subcellular location prediction, and even GO number. The program in the RIGD will search the database for eligible intronless genes and list them, and then users can click to view the detailed information. In addition, the RIGD will renumber the intronless genes after processing, and the rule is the abbreviation of the species name + “IG” + chromosome number + the order number of the gene (starting from 1). The gene number in the original data is still retained, and either the RIGD number or the gene number of the original data can be used for searching (Figure 5).

### Blast

**FIGURE 6:**
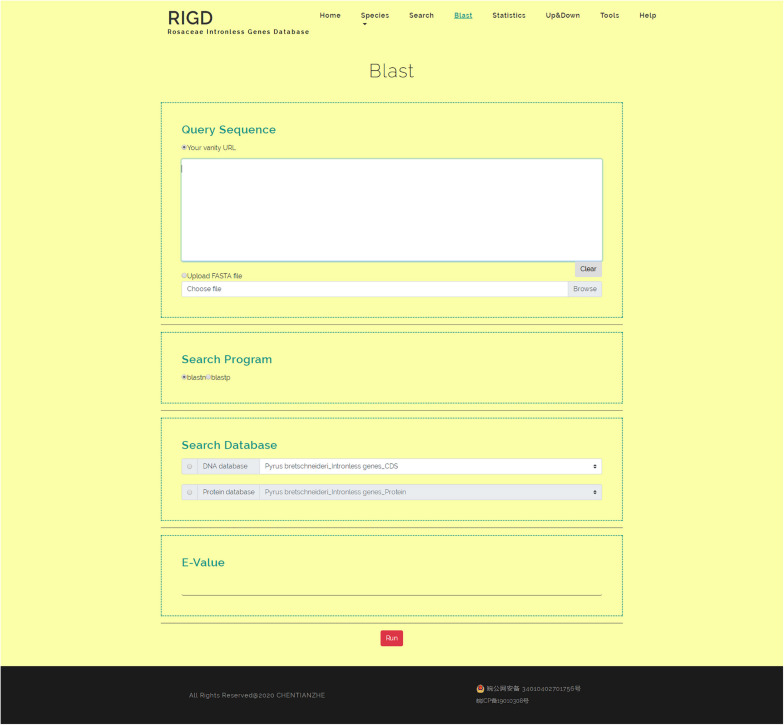
The website page of Blast. The interface can be used for Blast comparison of intronless genes in RIGD, researchers can use blastp/blastn to analyze, and the E- value can be set.

The RIGD has Blast software installed on the server, moreover, the intronless gene CDS and protein sequences of the six Rosaceae species stored in the RIGD were formatted into the Blast local database. In the Blast interface, users can paste a sequence or upload a fasta-format file to match with the RIGD’s local Blast database and find the putative homologous sequences of these intronless genes in different species. The databases can be compared (CDS/protein), and Blast programs (Blastn/Blastp) and e-values can all be selected or entered into the interface (Figure 6).

### Statistics

The results of comparative analysis among the six species are shown on the Statistics interface with statistical charts. Four pictures investigate the general trends in protein length distribution, show us the distribution of pI, the distribution of Mw and the statistics of subcellular localization.

### Upload and Download

In the interface, according to the species name and the chromosome number in each species, users can download the following data in the “Download” section, namely, the CDS and protein sequence of intronless genes, the prediction of pI and Mw, the results of the sequence compared with the nr database, the results of protein domain analysis, the results of subcellular localization prediction, the results of protein function prediction, and the result of GO analysis, according to the species name and the chromosome number in each species. In the “Upload” section, users can upload intronless gene sequence files of other species or analysis result files to the RIGD server to expand the RIGD in the future.

### Tools

We designed the Tools interface to collect some tools for intronless gene analysis or other practical bioinformatics analysis that will be developed in the future. The tool now available on this interface is a program that can batch submit sequences to ExPASy for pI/Mw prediction.

### Contact Us

The Contact us interface is divided into “Contact us” and “Links.” Users can email the RIGD’s administrator in the “Contact us” interface to ask any questions or provide valuable suggestions. The “Links” interface contains links to external databases and analysis tools that the RIGD references.

## Case Study

### The Results of Comparative Analysis Among the Six Species in Rosaceae

**FIGURE 7:**
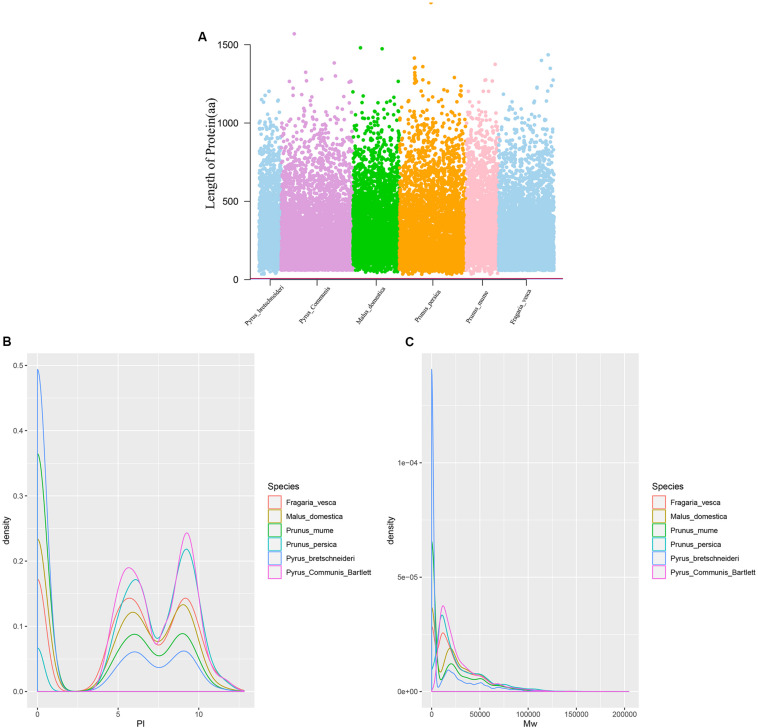
**(A)** The length of protein (number of amino acids). Most proteins were less than 500 aa in length. **(B)** The distribution of pI. The graphic shows three peaks, their pI close to 0.6 and 8. **(C)** The distribution of Mw. Most predicted protein molecular weights were less than 100000 Da.

**FIGURE 8:**
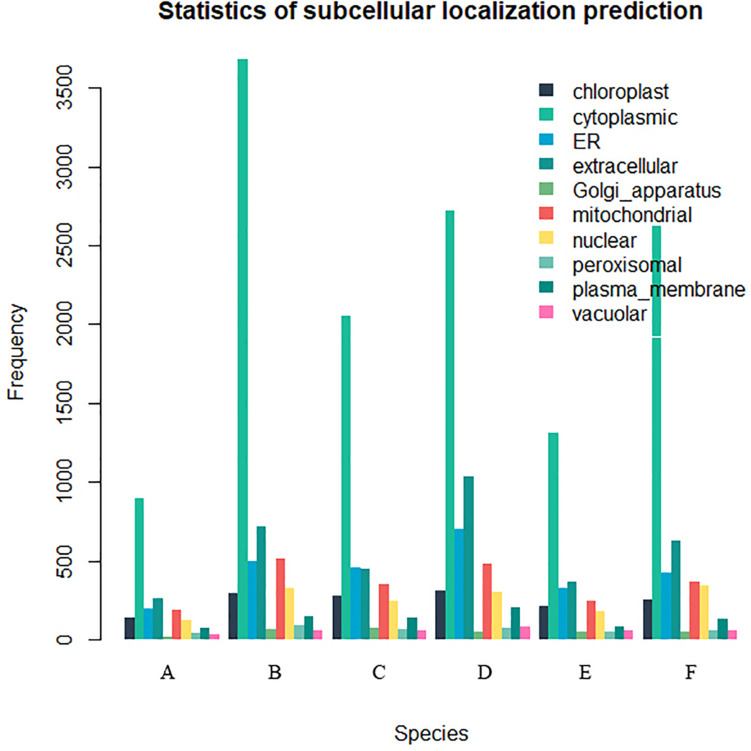
The statistics of subcellular localization prediction. The largest number of intronless genes were categorized as cytoplasmic, extracellular also accounted for a large proportion. **(A)** *Pyrus bretschneideri*. **(B)** *Pyrus Communis*. **(C)** *Malus domestica*. **(D)** *Prunus persica*. **(E)** *Prunus mume*. **(F)** *Fragaria vesca.*

**FIGURE 9:**
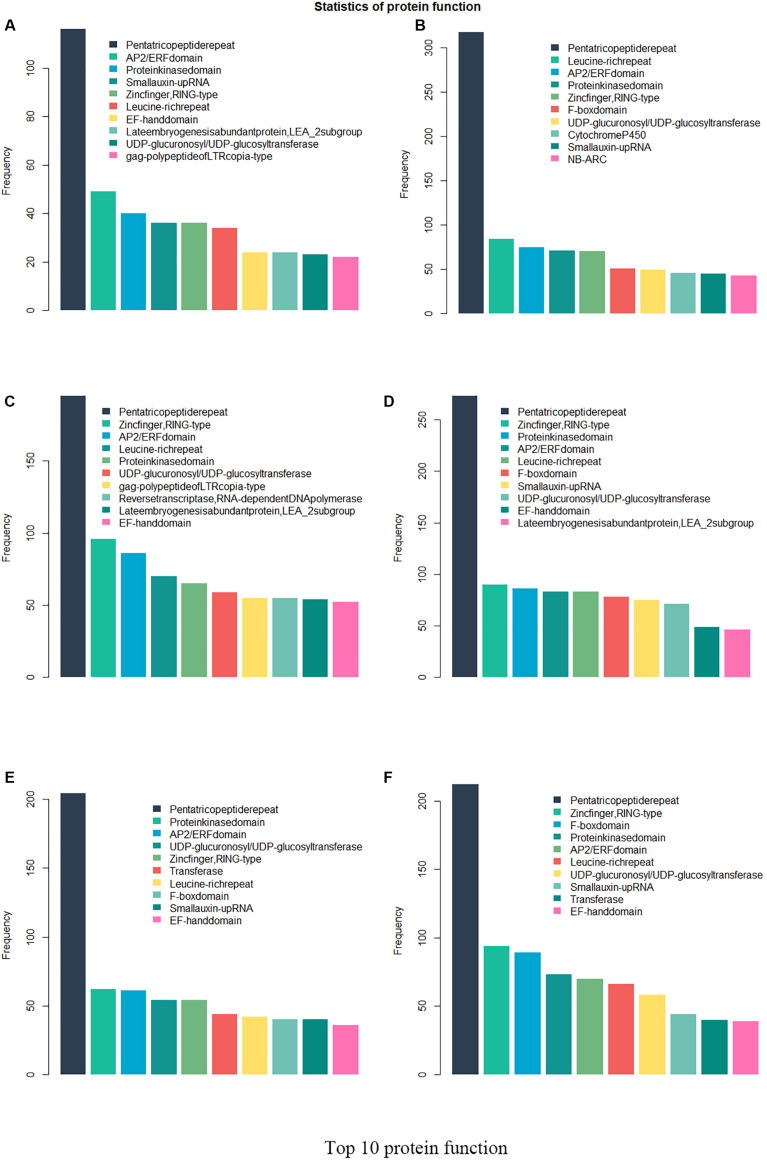
Top 10 largest number of intronless genes in protein function. The largest number of intronless genes were predicted for pentatricopeptide repeat. AP2/ERF domain in *Pyrus bretschneideri*, Leucine-rich repeat in *Pyrus communis*, Zinc finger (RING-type) in *Malus domestica*, *Prunus persica* and *Fragaria vesca*, and protein kinase domain in *Prunus mume* accounted for a large proportion. **(A)** *Pyrus bretschneideri*. **(B)** *Pyrus communis*. **(C)** *Malus domestica*. **(D)** *Prunus persica*. **(E)** *Prunus mume*. **(F)** *Fragaria vesca.*

Twenty-six thousand two hundred sixty intronless genes were identified from six Rosaceae species. *Pyrus bretschneideri*, *Pyrus communis*, *Malus domestica*, *Prunus persica*, *Prunus mume*, and *Fragaria vesca* consist of 5.44% (1966), 17.79% (6388), 10.38% (4143), 22.20% (5951), 12.97% (2882), and 17.35% (4930) intronless genes, respectively (Table 2). The distribution of intronless genes on chromosomes was uneven in different species (Supplementary Figures 1–6). Although the number of intronless genes varied greatly from chromosome to chromosome, the proportion of intronless genes on each chromosome did not vary much among species (Supplementary Figure 7). The average protein length was ∼333.4 amino acids (aa) in *Pyrus bretschneideri*, 258.7 aa in *Pyrus communis*, 321.4 aa in *Malus domestica*, 277.5 aa in *Prunus persica*, 351.5 aa in *Prunus mume*, and 275.0 aa in *Fragaria vesca* (Figure 7A). The distribution of pI had three peaks (Figure 7B), and the distribution of Mw gathered at the front of the diagram, most predicted protein molecular weights were less than 100000 Da (Figure 7C). The largest number of intronless genes were categorized as cytoplasmic in their cellular role (Figure 8). The largest number of intronless genes in six species were predicted for pentatricopeptide repeat in their protein function. The second largest number of intronless genes were predicted for AP2/ERF domain in *Pyrus bretschneideri*, Leucine-rich repeat in *Pyrus communis*, Zinc finger (RING-type) in *Malus domestica*, *Prunus persica* and *Fragaria vesca*, and protein kinase domain in *Prunus mume*. Top 10 largest number of intronless genes in protein function were shown in Figure 9. The largest number of intronless genes were classified as biological process in GO categories. The largest proportion of intronless genes in six species were classified as cell and cell part (Table 3 and Supplementary Figures 8–13).

**TABLE 2:** The number of intronless genes reported for each species.

**Species**	**Chromosome**	**Amount**	**Species**	**Chromosome**	**Amount**	**Species**	**Chromosome**	**Amount**
*Pyrus bretschneideri*	Chr1	99	*Pyrus communis*	Chr1	298	*Malus domestica*	Chr1	191
	Chr2	107		Chr2	384		Chr2	246
	Chr3	147		Chr3	345		Chr3	260
	Chr4	118		Chr4	303		Chr4	195
	Chr5	138		Chr5	493		Chr5	292
	Chr6	104		Chr6	324		Chr6	185
	Chr7	151		Chr7	431		Chr7	247
	Chr8	76		Chr8	302		Chr8	185
	Chr9	105		Chr9	273		Chr9	243
	Chr10	150		Chr10	451		Chr10	300
	Chr11	107		Chr11	435		Chr11	270
	Chr12	91		Chr12	340		Chr12	210
	Chr13	96		Chr13	383		Chr13	254
	Chr14	99		Chr14	291		Chr14	224
	Chr15	179		Chr15	558		Chr15	345
	Chr16	75		Chr16	350		Chr16	245
	Chr17	124		Chr17	427		Chr17	251
	Total	1966		Total	6388		Total	4143
*Prunus persica*	Chr1	1272	*Prunus mume*	Chr1	399			
	Chr2	727		Chr2	570	*Fragaria vesca*	Chr1	523
	Chr3	709		Chr3	371		Chr2	708
	Chr4	643		Chr4	364		Chr3	811
	Chr5	517		Chr5	338		Chr4	640
	Chr6	855		Chr6	338		Chr5	672
	Chr7	577		Chr7	259		Chr6	997
	Chr8	651		Chr8	243		Chr7	579
	Total	5951		Total	2882		Total	4930

**TABLE 3:** The number of intronless genes in GO categories.

**Species**	**Annotated Genes**	**GO Terms**			
		**Biological**	**Cellular**	**Function**	**Total**
*Pyrus bretschneideri*	780	666	630	539	1835
*Pyrus communis*	2550	2249	2140	1907	6296
*Malus domestica*	1720	1474	1385	1219	4078
*Prunus persica*	1805	1548	1479	1272	1299
*Prunus mume*	1151	983	941	840	2764
*Fragaria vesca*	1638	1429	1348	1203	3980

### Analysis of Intronless Pentatricopeptide Repeat Gene Family in *Pyrus bretschneideri*

In *Pyrus bretschneideri*, the largest intronless gene family is the Pentatricopeptide Repeat gene family. Meanwhile, PPR gene family is also one of the largest families found in most plants, which plays a wide and crucial role in plant growth and development. We searched RIGD database by using Pfam ID of Pentatricopeptide Repeat gene family (PF01535, PF13041, and PF13812), the predicted protein function was used to determine whether it belonged to PPR gene. The analysis results of isoelectric point, protein molecular weight and subcellular localization were obtained from RIGD by using the search interface. We downloaded the protein sequence, used the MEME SUITE (Bailey et al., 2009) and TBtools (Chen et al., 2020) to analysis the motif of intronless PPR gene in *Pyrus bretschneideri*.

**FIGURE 10:**
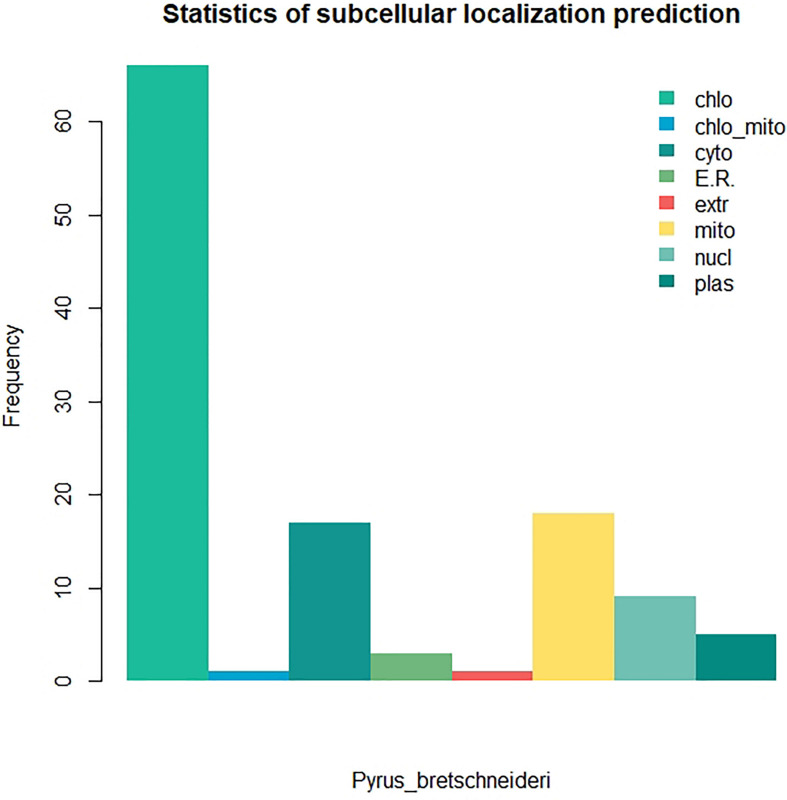
Subcellular localization prediction of intronless PPR genes in *Pyrus bretschneideri*. Most proportion of genes were located in chloroplasts.

**FIGURE 11:**
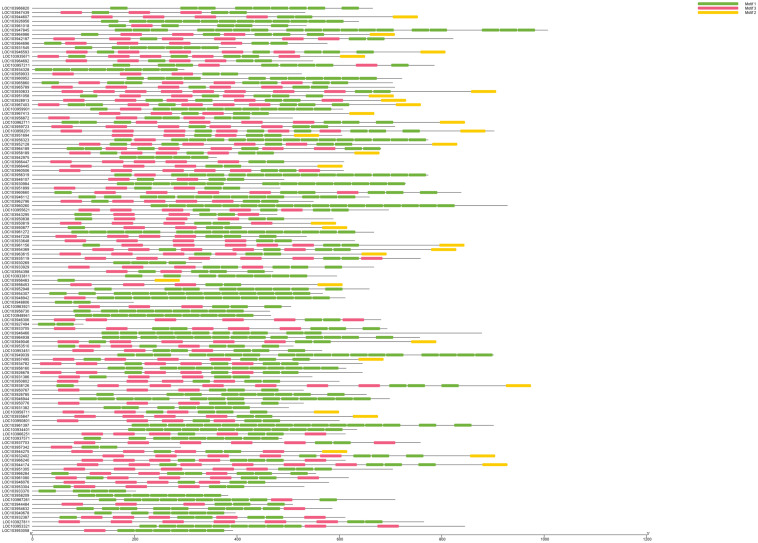
Motif analysis and visualization. Motif1 is the most covered. Motif3 only existed at the end of amino acid sequence. LOC103956483 was the only one of the 120 intronless PPR genes contained only Motif1 and Motif2.

We identified 120 intronless PPR genes in *Pyrus bretschneideri*. The relative molecular weight of each protein was between 11.5 and 113.7 kD. The molecular weight of gene named LOC103927494 was the smallest, while the molecular weight of LOC103947845 was far higher than that of other genes, 10 times the minimum molecular weight, and more than twice the average molecular weight of 120 amino acid sequences. In addition, the predicted results of theoretical isoelectric points were shown between 5.2 and 9.47. The isoelectric point of 46.3% members was less than 7 and belonged to acidic protein, while the other 53.7% were all basic proteins (Supplementary Table 1). The results of subcellular localization prediction showed that most genes were located in chloroplasts, some genes were in mitochondria and cytoplasm, a few genes were in nucleus, plastids, endoplasmic reticulum and extracellular regions. The above results showed that intronless PPR genes still has the characteristic of typical localization in semi-autonomous organelles, which was consistent with the localization of PPR protein in other plants (Figure 10). We identified three sequence motif: Motif1 (GIKPDVEHYGCMVDLLGRAGRLEEAEELIKEMPFK), Motif2 (IRVVKNLRVCGDCHSAIKLISKVVGREIIVRDANRFHHFKD GSCSCGDYW), and Motif3 (FVGNALIDMYAKCGSLEEARKV FDEMPERNVVSWNAMISGYAQ). Motif1 was covered in 120 intronless PPR genes, and was highly conserved. Thirty-three genes contained only Motif1 (27.5%), 58 genes contained Motif1 and Motif3 (48.3%) and 28 genes contained all three motif (23.3%). It is worth noting that Motif3 only existed at the end of amino acid sequence. In addition, LOC103956483 contained Motif1 and Motif2, which was the only one of the 120 intronless PPR genes contained only Motif1 and Motif2 (Figure 11).

## Discussion

In eukaryotes, there are intronless genes because there is no special structure of introns in genes, so studying the functions and evolutionary characteristics of these genes can help us to understand the evolution rules of related genes and genomes. Meanwhile, the exploration of intronless genes can help researchers to explore the effects of introns and selective splicing mechanisms on eukaryotes from the perspective of reverse thinking.

Because of the importance of intronless genes in comparative genomics and evolutionary biology, research on intronless genes in eukaryotes has been the focus of researchers for a long time. It is necessary to establish a centralized data platform for the integration, comparison, and analysis of the function and evolution of intronless genes on a larger scale. Little work has been done, as only a few databases exist, while Genome SEGE and IGDD have stopped providing services. IGD was limited to human intronless genes, which were annotated in different databases. PIGD focused on the intronless genes of Poaceae species and conducted a systematic comparative analysis from the perspective of comparative genomics, but the database has been damaged for providing retrieval services. As a result, users can only download the original data of the intronless gene sequences and the results of the analysis.

The RIGD, as the latest intronless gene database, integrates the intronless gene data of six species of Rosaceae and provides a systematic comparative analysis. The RIGD was designed as a simple, easy-to-use, and esthetically pleasing website interface that provides a feature-rich, user-friendly integrated data and analytics tool. The Species interface provides a download of the original data classified by chromosome number and analysis methods. The Statistics interface presents the results of systematic comparative genomics analysis of six species in the form of graphs. The Search interface allows users to search for data on intronless genes of interest. In addition, NCBI Blast, a common bioinformatics tool, is embedded in the RIGD to help researchers annotate new sequences and predict homology with genes in the RIGD. The RIGD also provides multiple interactive platforms, including Up&Down, Contact us and Links. Through these platforms, users can learn about the RIGD’s analytical methods, download data of interest, and upload their important scientific findings to facilitate communication and data sharing among researchers in the same research field.

The RIGD is built on a Tencent cloud server with stable service and convenience for long-term maintenance and updating. In the future, we hope to update and expand the RIGD by communicating with researchers. The number of species collected is expected to increase, and more detailed annotation information on intronless genes, such as spatio-temporal expression data of intronless genes in different growth stages and tissues of plants, homologous genes in the genome, metabolic pathways of genes, and more, are expected to be added. This information will allow researchers to further explore the function and evolutionary mechanisms of intronless genes. Moreover, we are also committed to developing powerful comparative analysis tools to make the RIGD a centralized platform for intronless gene information and analysis, enabling researchers to use the database for data mining and analysis in various aspects.

## Conclusion

With the development of sequencing technology, an increasing number of plant genomes are sequenced and annotated, and there will be increasing data regarding intronless genes in the future. It is feasible to integrate, compare and analyze the function and evolution of intronless genes in a wide range. We developed the RIGD platform, collected and systematically analyzed the data from intronless genes in six species of Rosaceae, and provided a series of tools for users to search the data of intronless genes of interest and communicate with us. With the support of researchers, we eventually hope to develop a platform for integrating data from eukaryotic intronless genes with tools for comparative genomics analysis, which can greatly promote the research of intronless genes in plants, thus mining valuable genomic resources and helping researchers find more interesting discoveries.

## Data Availability Statement

Publicly available datasets were analyzed in this study. This data can be found here: ftp://ftp.bioinfo.wsu.edu/www.rosaceae.org/Pyrus_x_bretschneideri/Pbretschneideri-genome.v1.1, ftp://ftp.bioinfo.wsu.edu/species/Pyrus_communis/Pcommunis_DH_genome.v2.0, ftp://ftp.ncbi.nlm.nih.gov/genomes/all/GCF/002/114/115/GCF_002114115.1_ASM211411v1, ftp://ftp.bioinfo.wsu.edu/species/Prunus_persica/Prunus_persica-genome.v2.0.a1, ftp://ftp.ncbi.nlm.nih.gov/genomes/all/GCF/000/346/735/GCF_000346735.1_P.mume_V1.0, and ftp://ftp.bioinfo.wsu.edu/species/Fragaria_vesca/Fvesca-genome. v4.0.a1.

## Author Contributions

TC projected the study, constructed the platform, wrote programs in the website background and analysis, involved in the bioinformatics analysis, and drew up the manuscript. DM put into effect the mainly bioinformatics analysis, handled figures and tables, participated in the design of platform, and update of the database. XL, XC, HW, QJ, and XX collected and collated the data, helped with the design and update of the database, provided suggestions, and criticisms for improving the manuscript and website. YCo and YCi participated in the design, helped in writing the manuscript, and supervised the whole project. All authors read and accepted the final manuscript.

## Conflict of Interest

The authors declare that the research was conducted in the absence of any commercial or financial relationships that could be construed as a potential conflict of interest.

## Footnotes

1 http://www.rigdb.cn/

2 ftp://ftp.ncbi.nlm.nih.gov/genomes/all/

3 https://www.rosaceae.org/

4 https://www.mathworks.com/help/bioinfo/ref/chromosomeplot.html

5 ftp://ftp.ncbi.nlm.nih.gov/blast/db/FASTA/nr.gz

6 https://github.com/bbuchfink/diamond

7 https://bioperl.org/

8 https://web.expasy.org/compute_pi/

9 https://github.com/KohlbacherLab/MultiLoc2
