# Supplementary material for: RIGD: A Database for Intronless Genes in the Rosaceae
Source: Front Genet. 2020 Aug 7;11:868. doi: 10.3389/fgene.2020.00868 (PMC7426402; doi:10.3389/fgene.2020.00868)
Supplement: Supplementary file 1 [file Data_Sheet_1.docx]

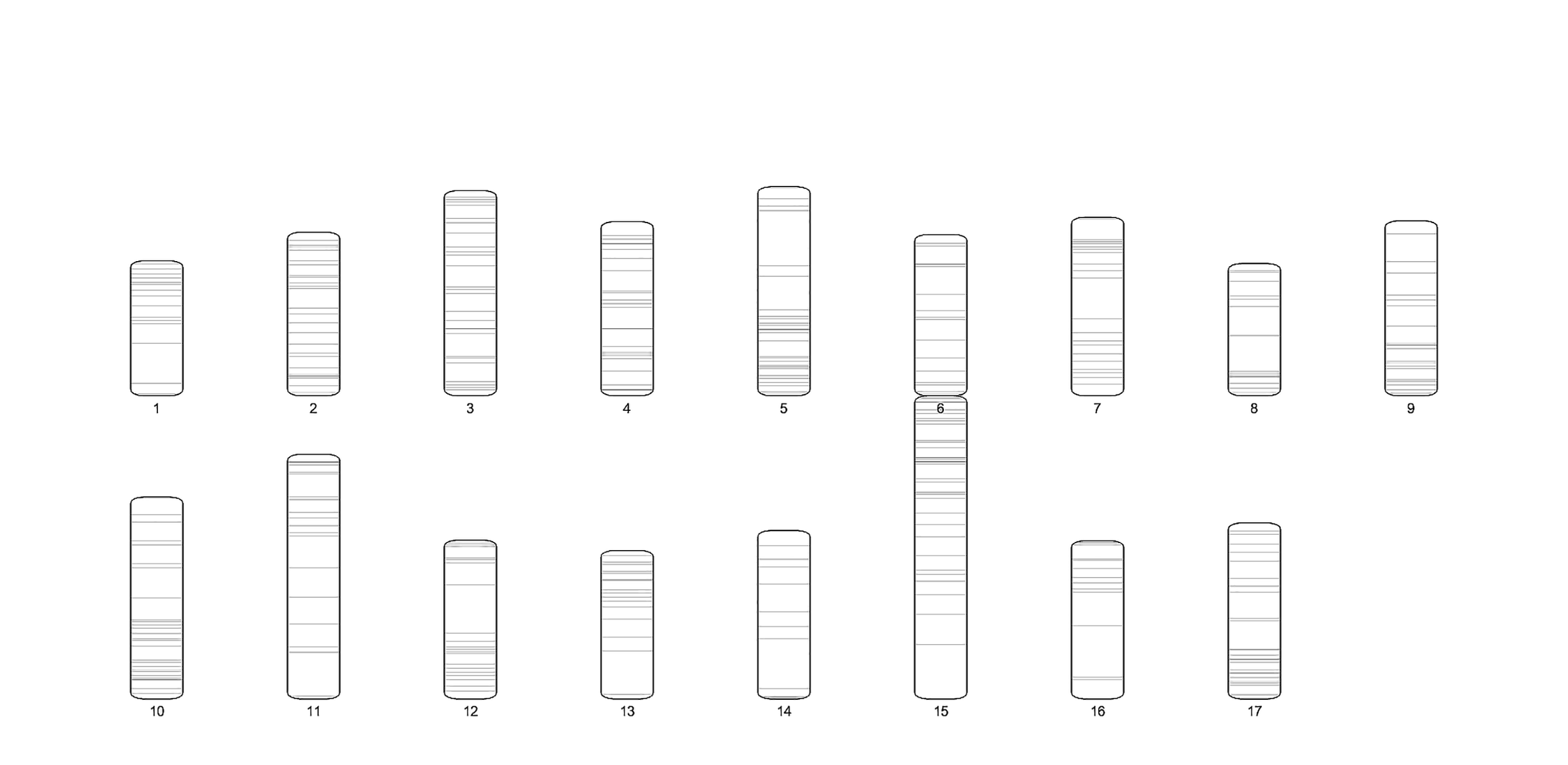


**Supplementary Fig. 1** The chromosome ideogram with intronless gene of *Pyrus bretschneideri.*

The distribution of intronless genes on chromosomes was uneven. The horizontal lines represent the location of genes.


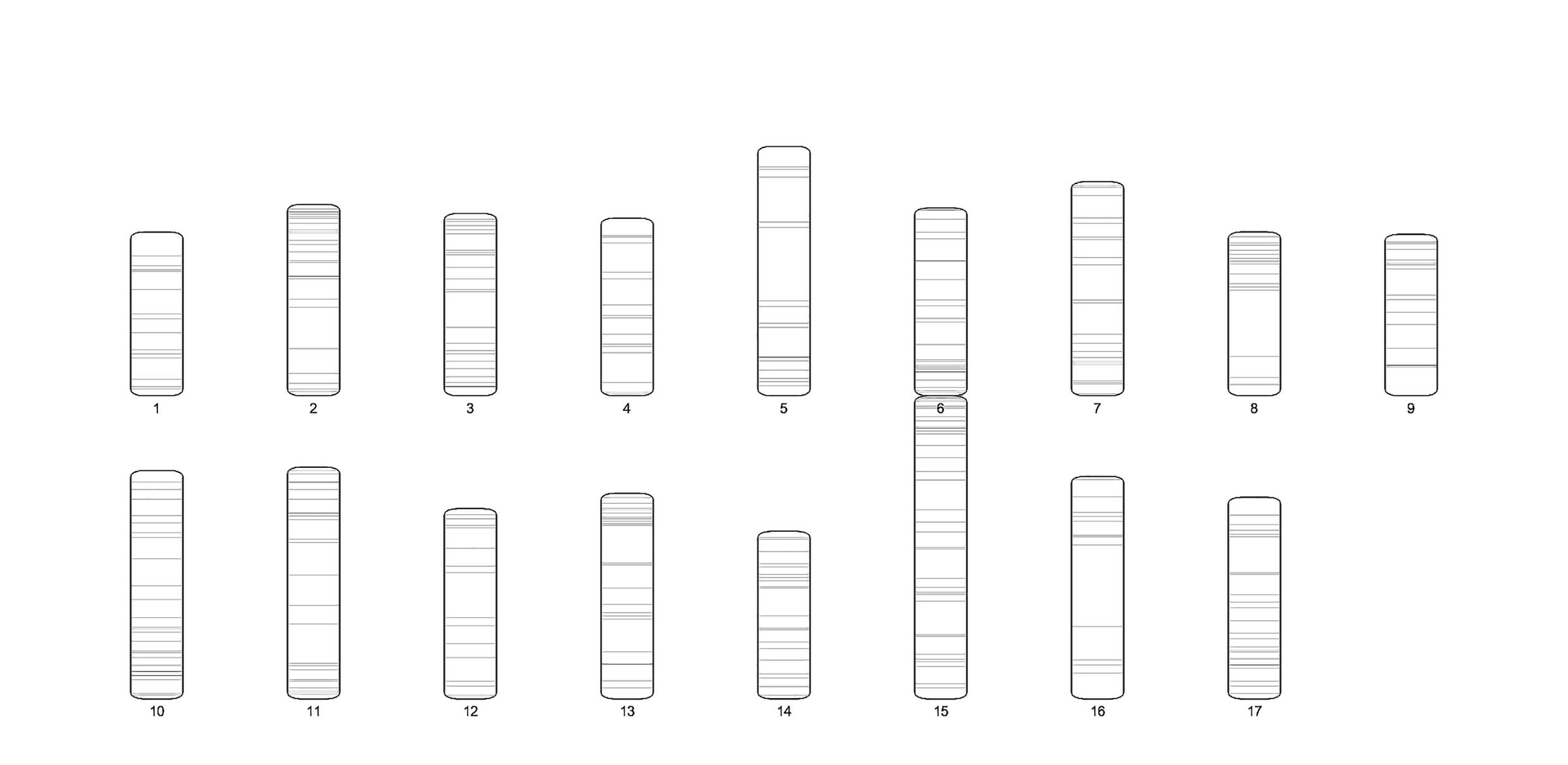


**Supplementary Fig. 2** The chromosome ideogram with intronless gene of *Pyrus Communis.*

The distribution of intronless genes on chromosomes was uneven. The horizontal lines represent the location of genes.


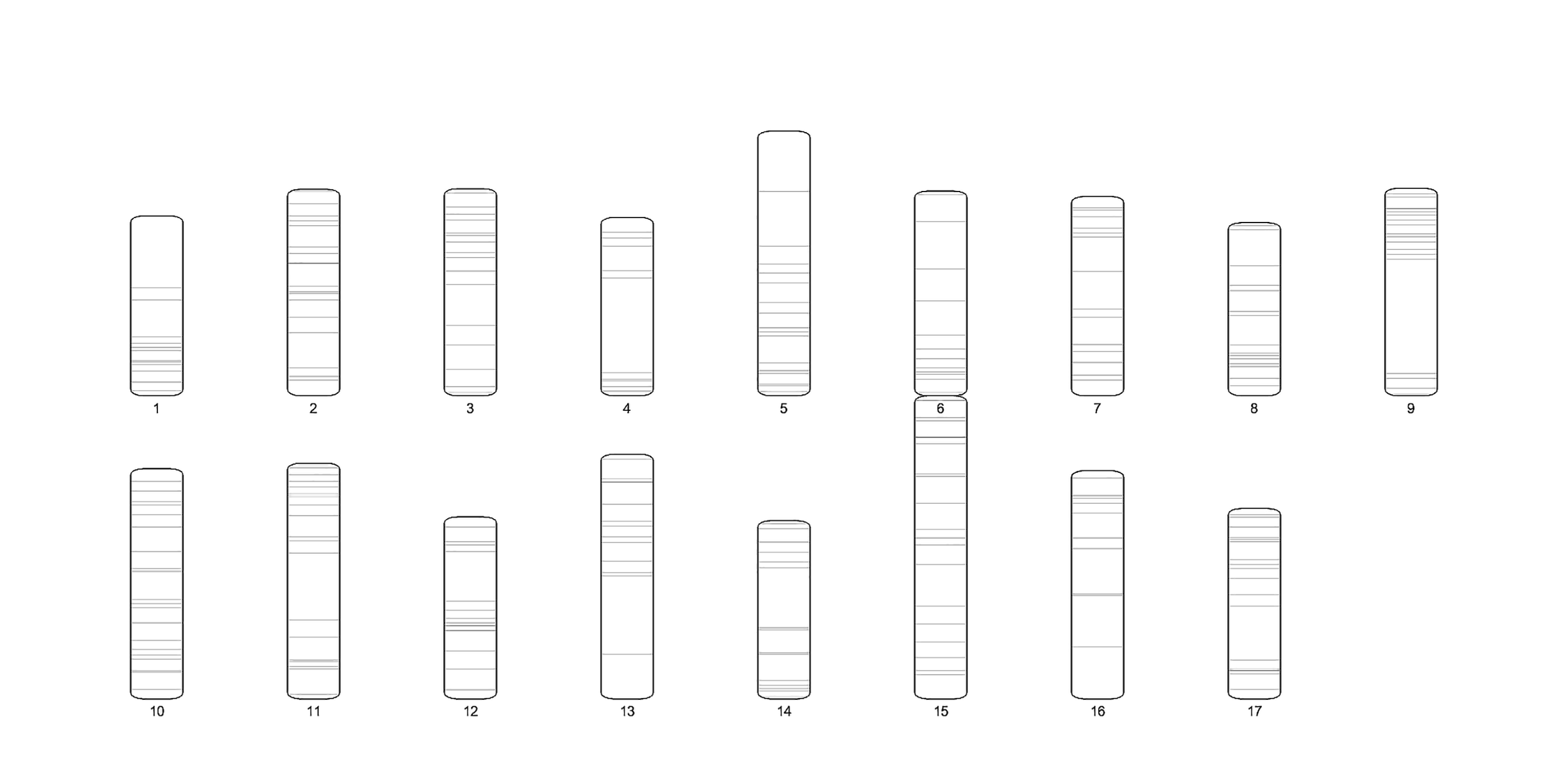


**Supplementary Fig. 3** The chromosome ideogram with intronless gene of *Malus domestica.*

The distribution of intronless genes on chromosomes was uneven. The horizontal lines represent the location of genes.


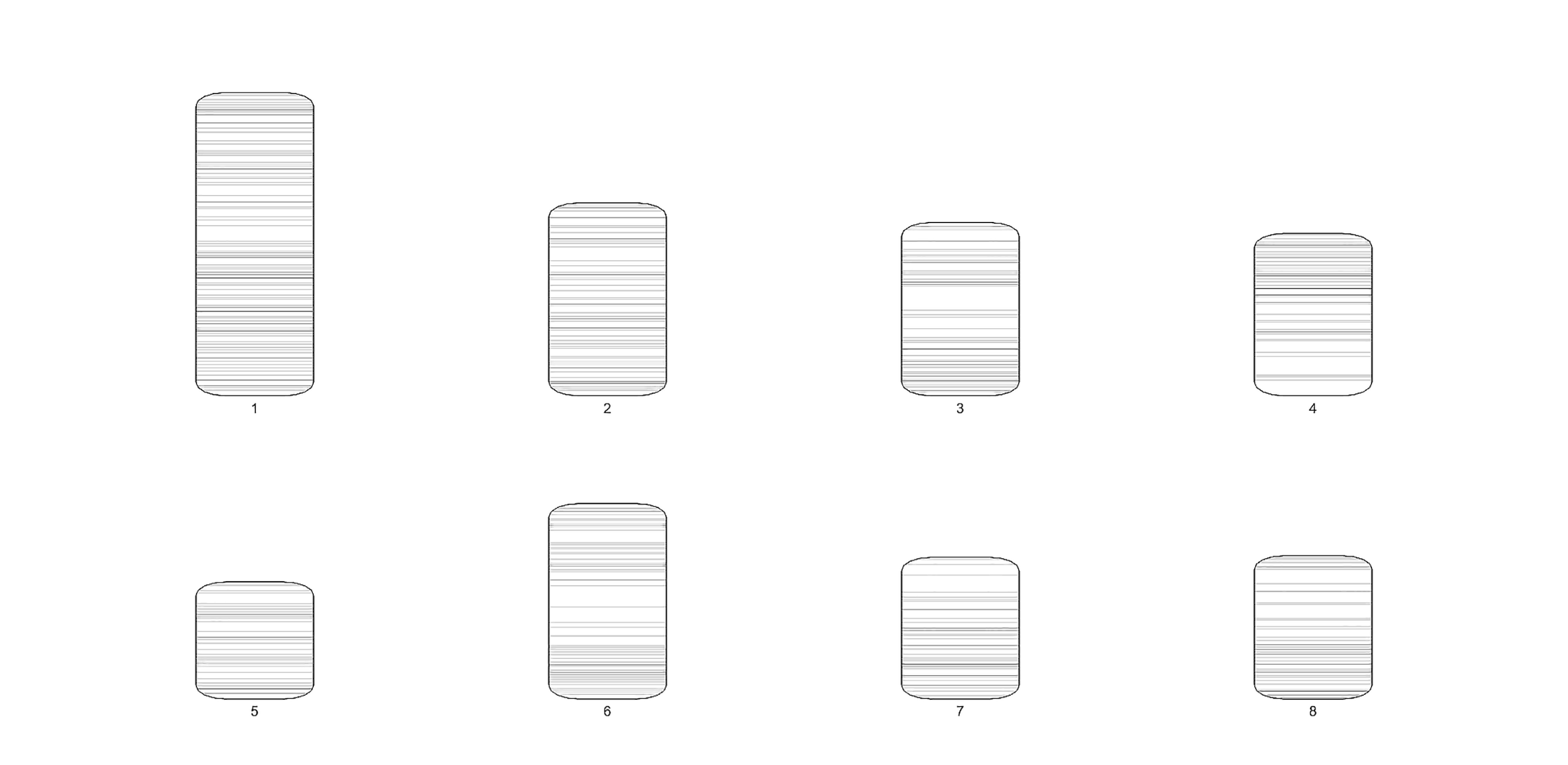


**Supplementary Fig. 4** The chromosome ideogram with intronless gene of *Prunus persica.*

The distribution of intronless genes on chromosomes was uneven. The horizontal lines represent the location of genes.


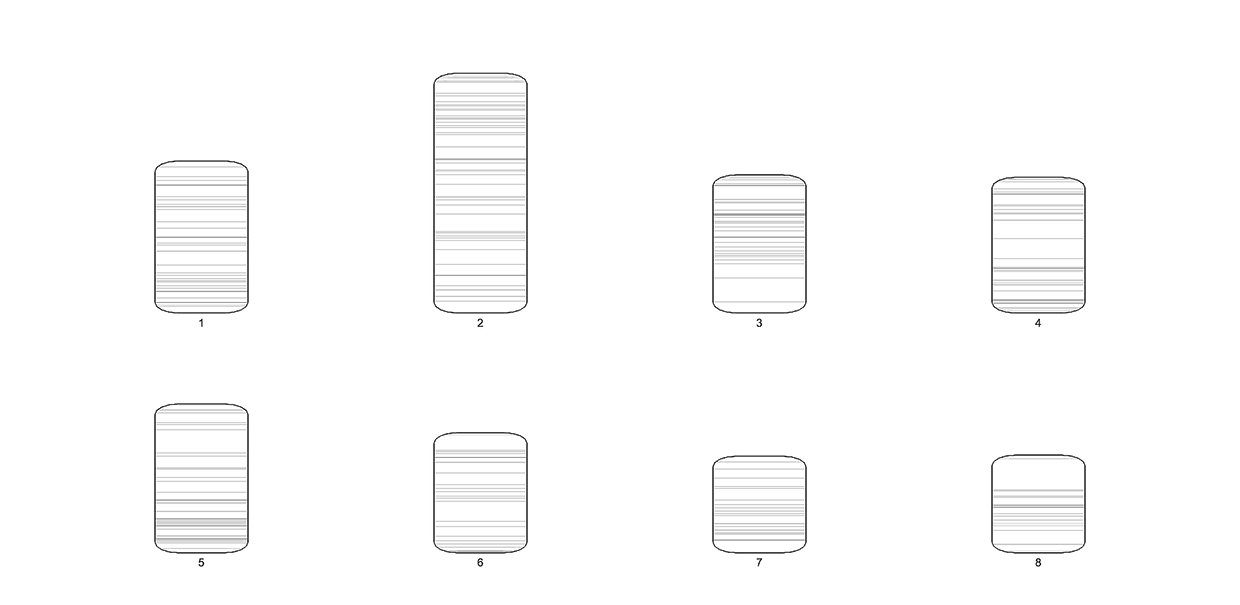


**Supplementary Fig. 5** The chromosome ideogram with intronless gene of *Prunus mume.*

The distribution of intronless genes on chromosomes was uneven. The horizontal lines represent the location of genes.


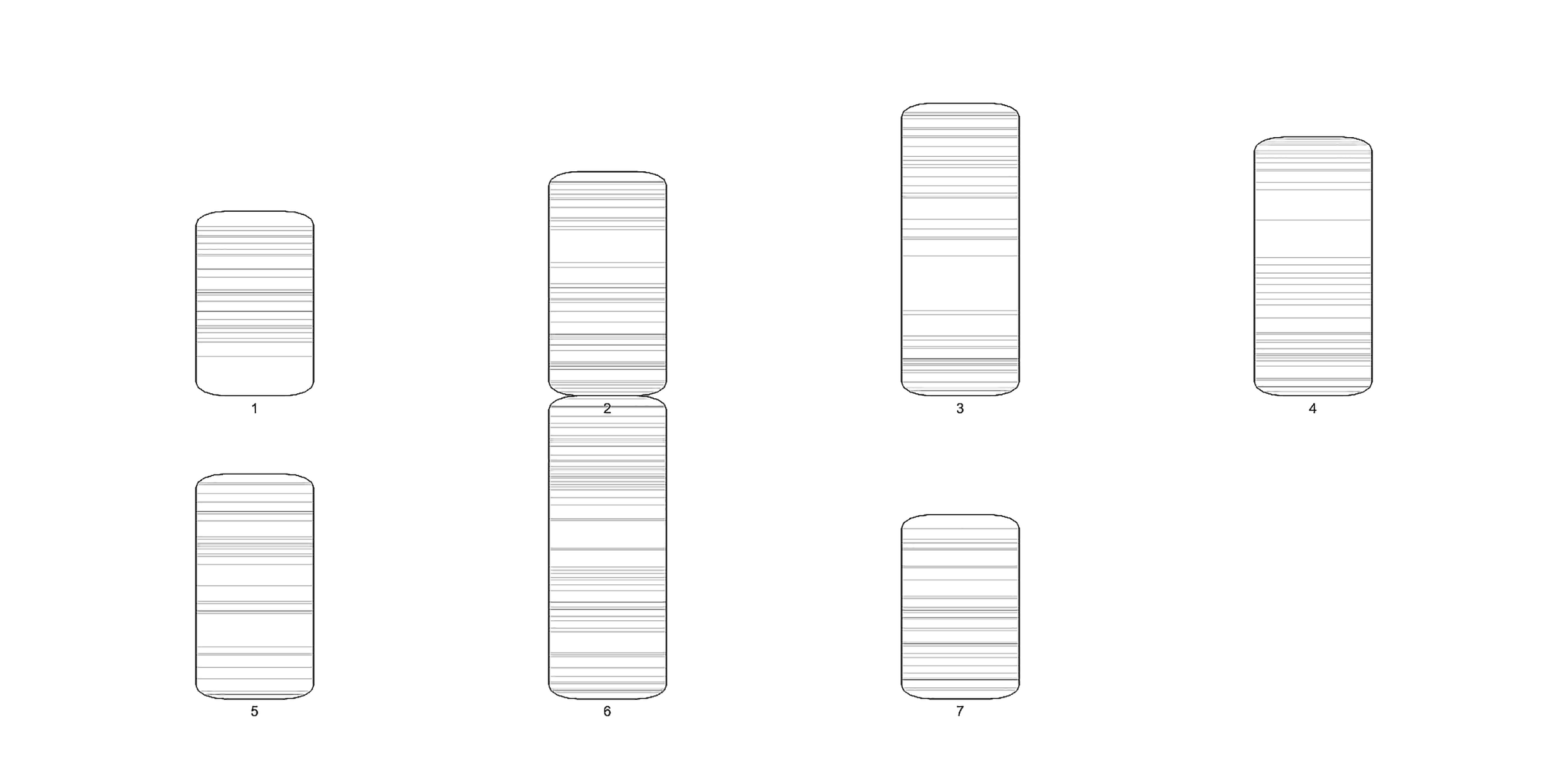


**Supplementary Fig. 6** The chromosome ideogram with intronless gene of *Fragaria vesca.*

The distribution of intronless genes on chromosomes was uneven. The horizontal lines represent the location of genes.


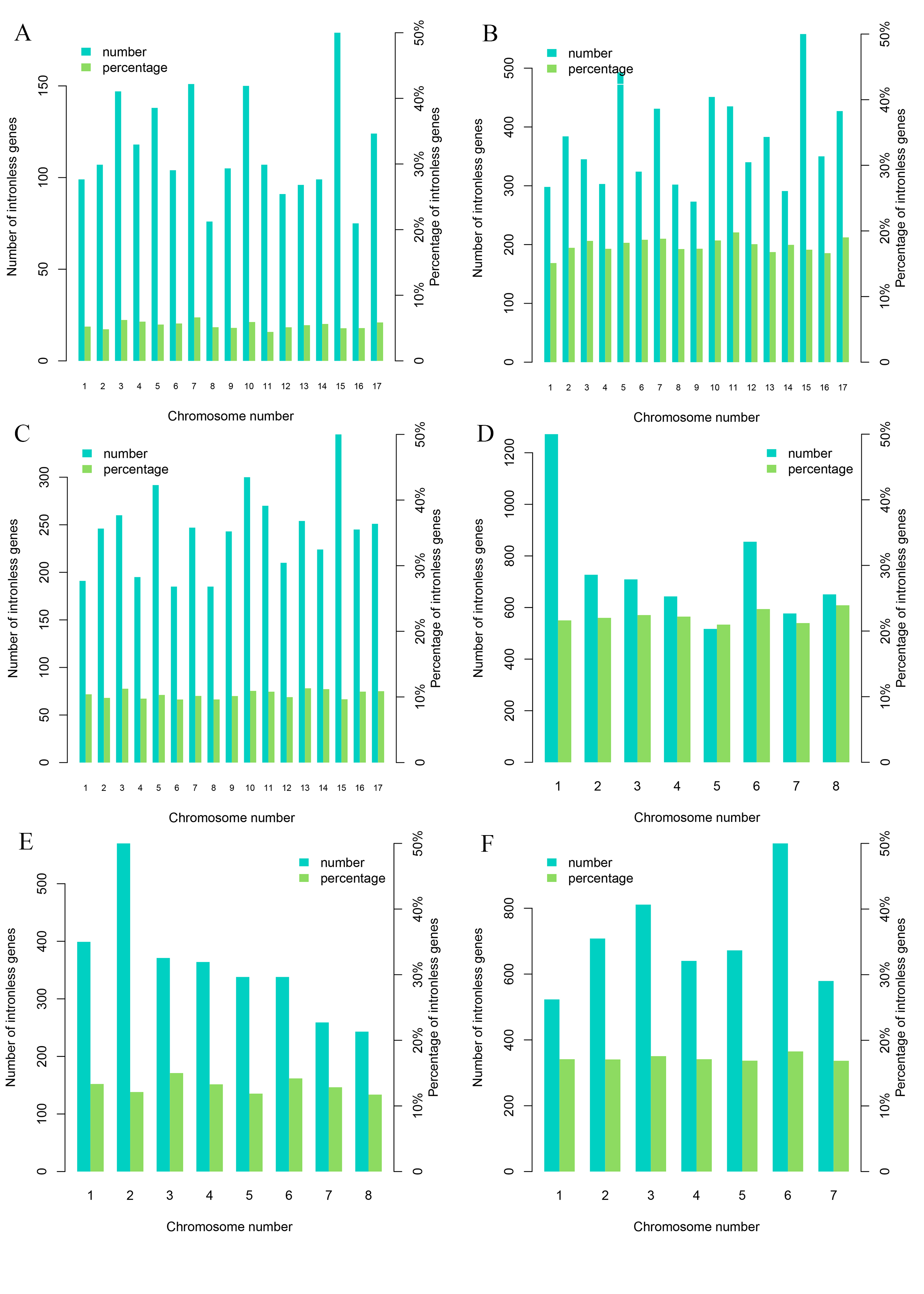


**Supplementary Fig. 7** The diagram of the number and proportion of intronless genes on each chromosome in six species. Although the number of intronless genes varied greatly from chromosome to chromosome, the proportion of intronless genes on each chromosome did not vary much among species

(a) *Pyrus bretschneideri* (b) *Pyrus Communis* (c) *Malus domestica* (d) *Prunus persica*

(e) *Prunus mume* (f) *Fragaria vesca*


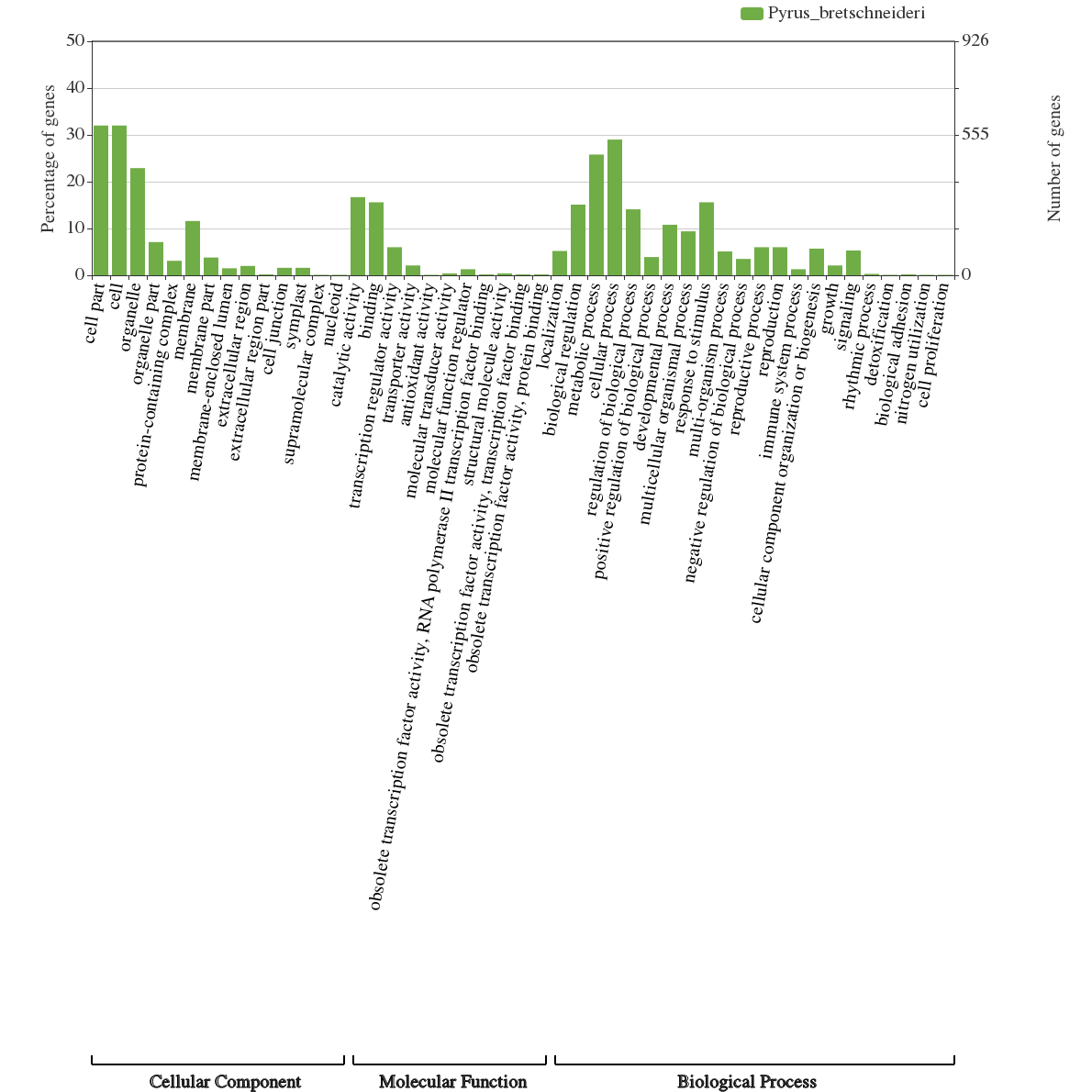


**Supplementary Fig. 8** The statistics of GO classifications in *Pyrus bretschneideri*. The largest proportion of intronless genes were classified as cell and cell part.


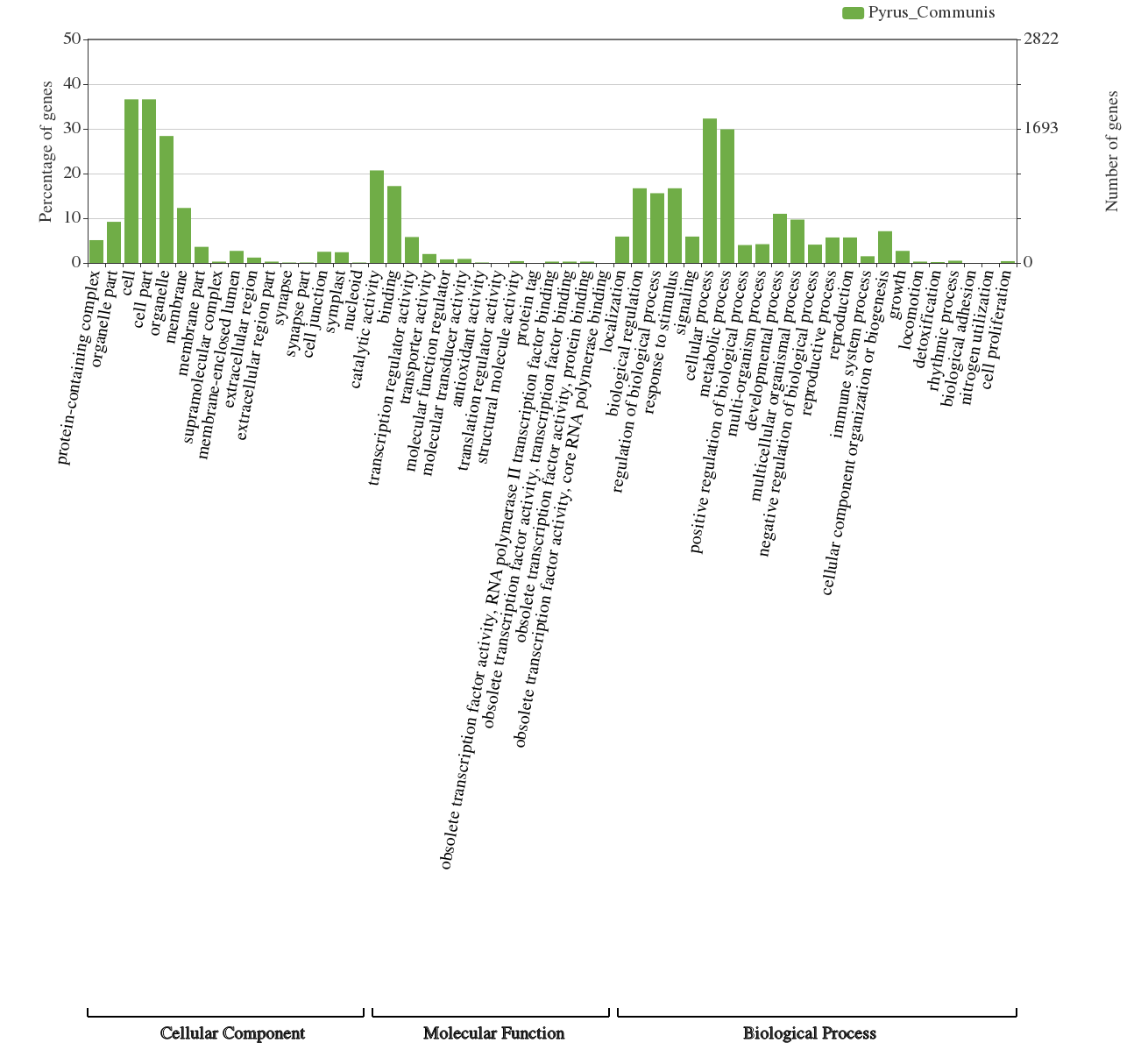


**Supplementary Fig. 9** The statistics of GO classifications in *Pyrus Communis.* The largest proportion of intronless genes were classified as cell and cell part.


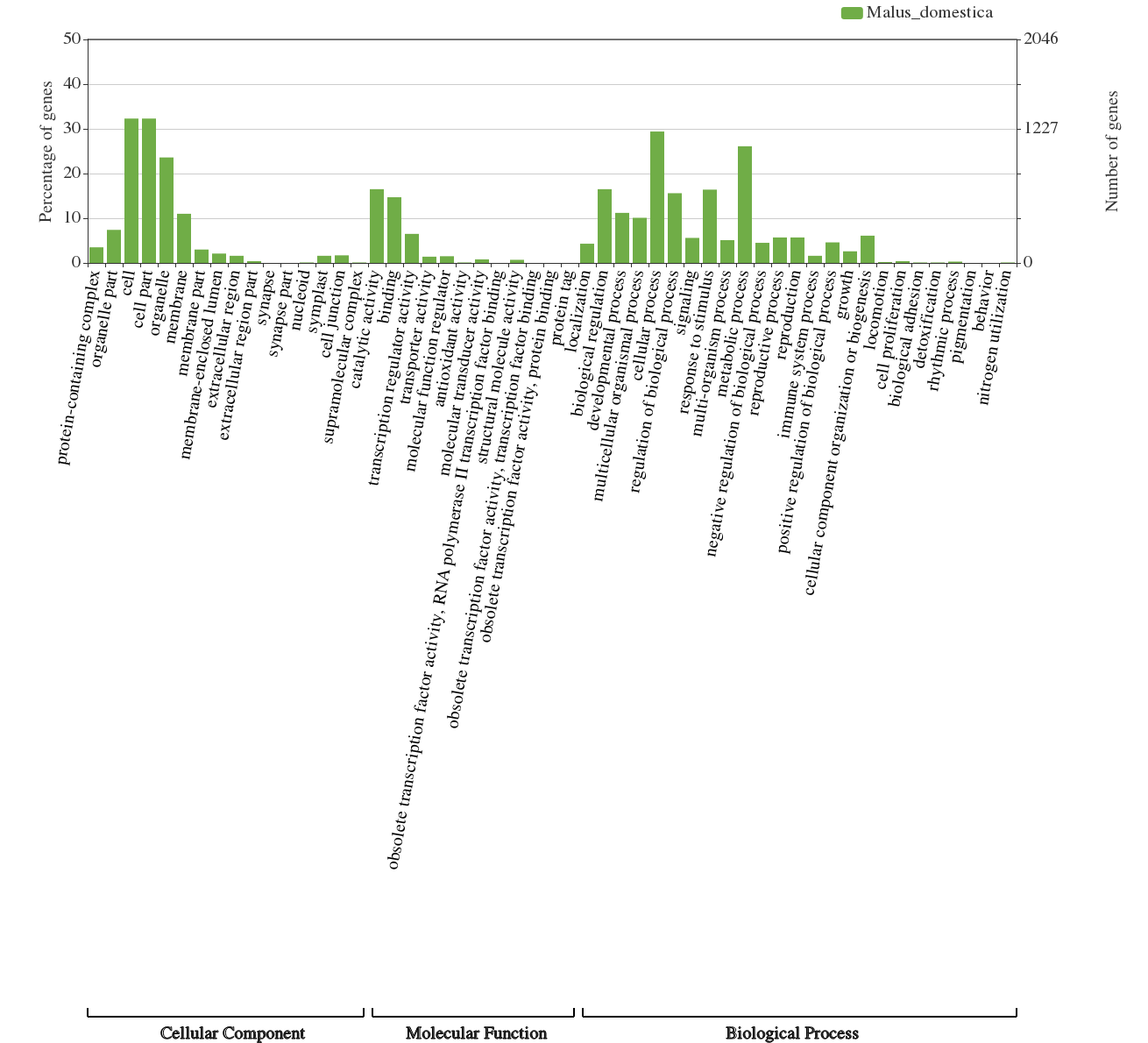


**Supplementary Fig. 10** The statistics of GO classifications in *Malus domestica*. The largest proportion of intronless genes were classified as cell and cell part.


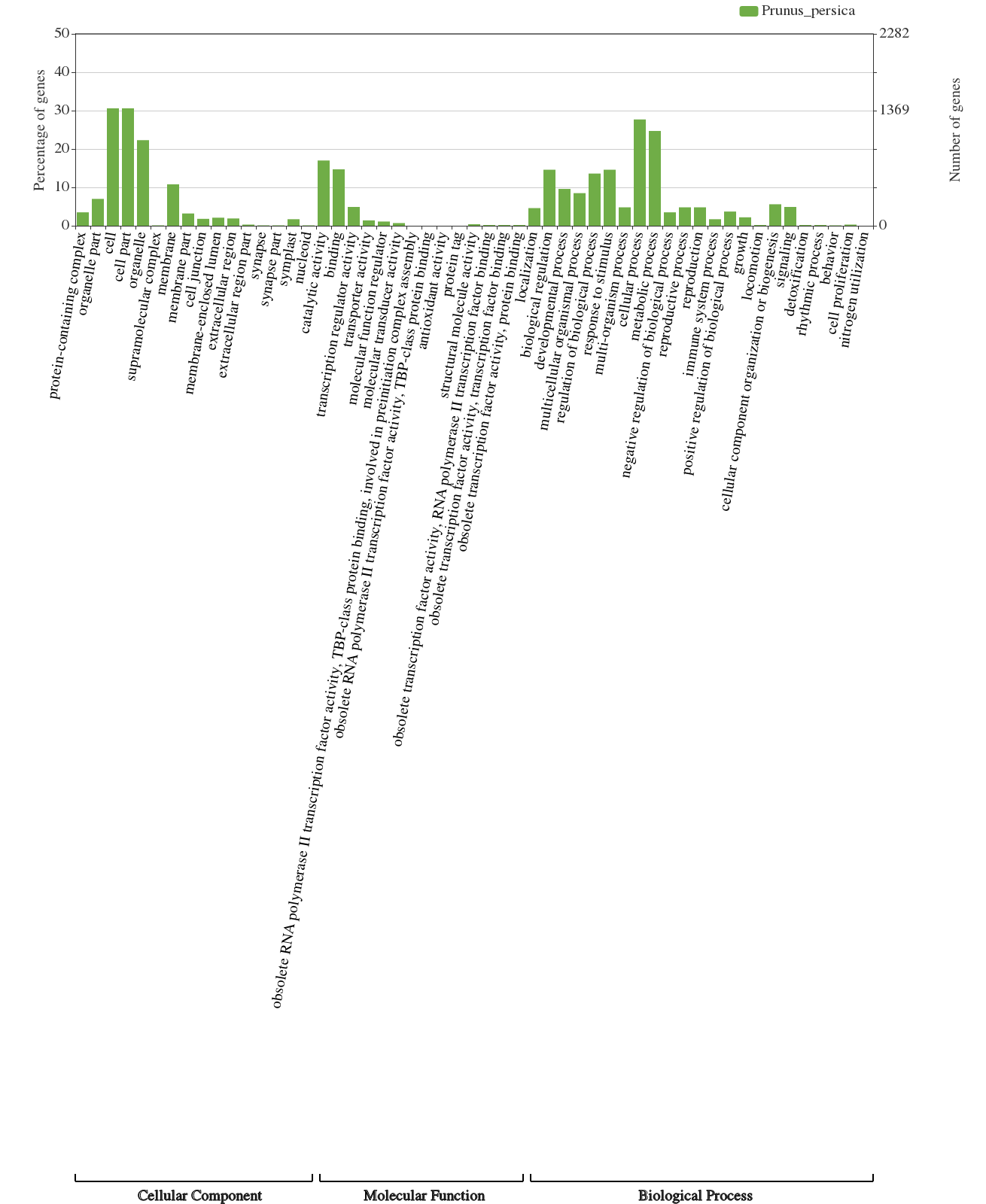


**Supplementary Fig. 11** The statistics of GO classifications in *Prunus persica*. The largest proportion of intronless genes were classified as cell and cell part.


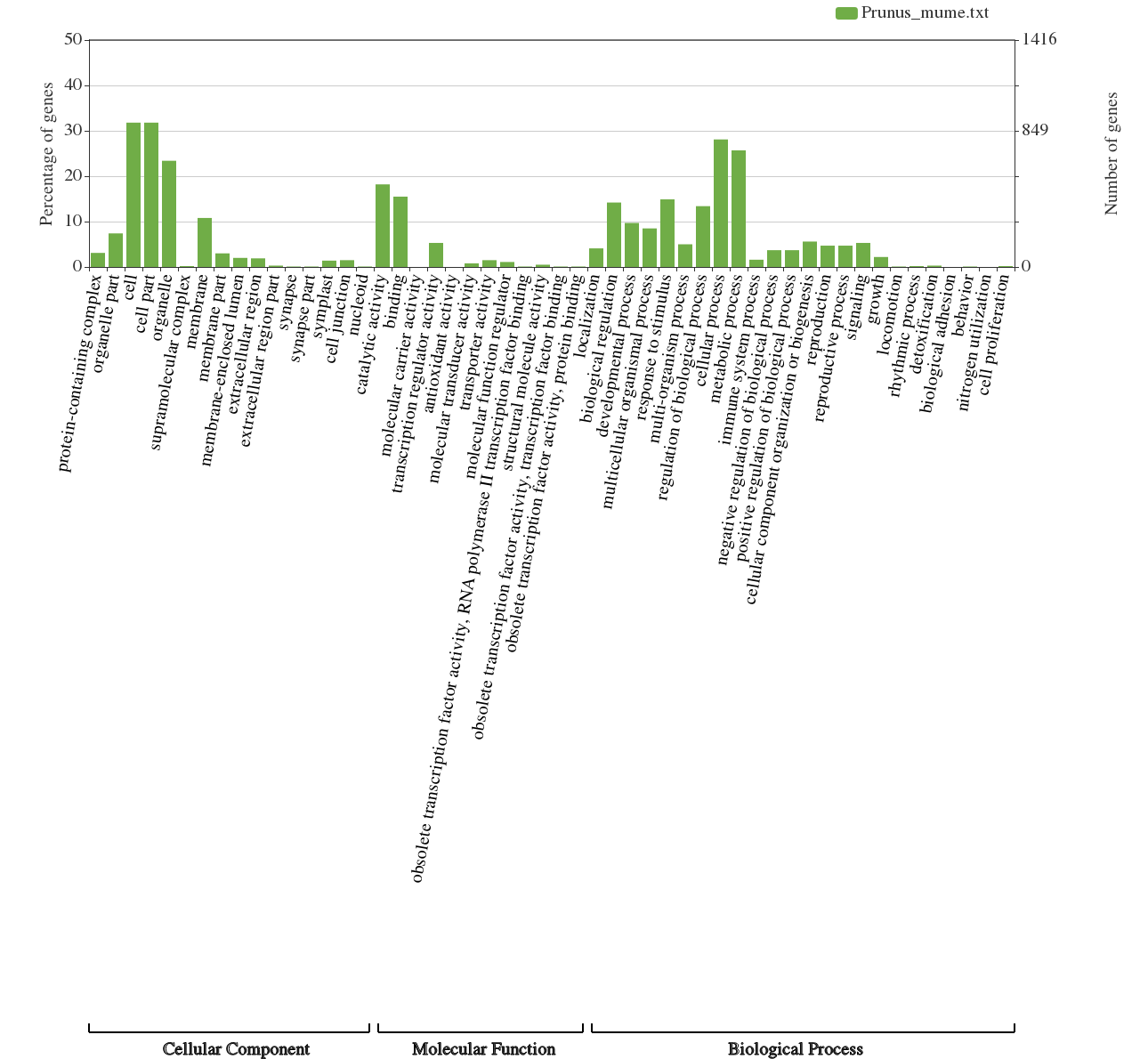


**Supplementary Fig. 12** The statistics of GO classifications in *Prunus mume*. The largest proportion of intronless genes were classified as cell and cell part.


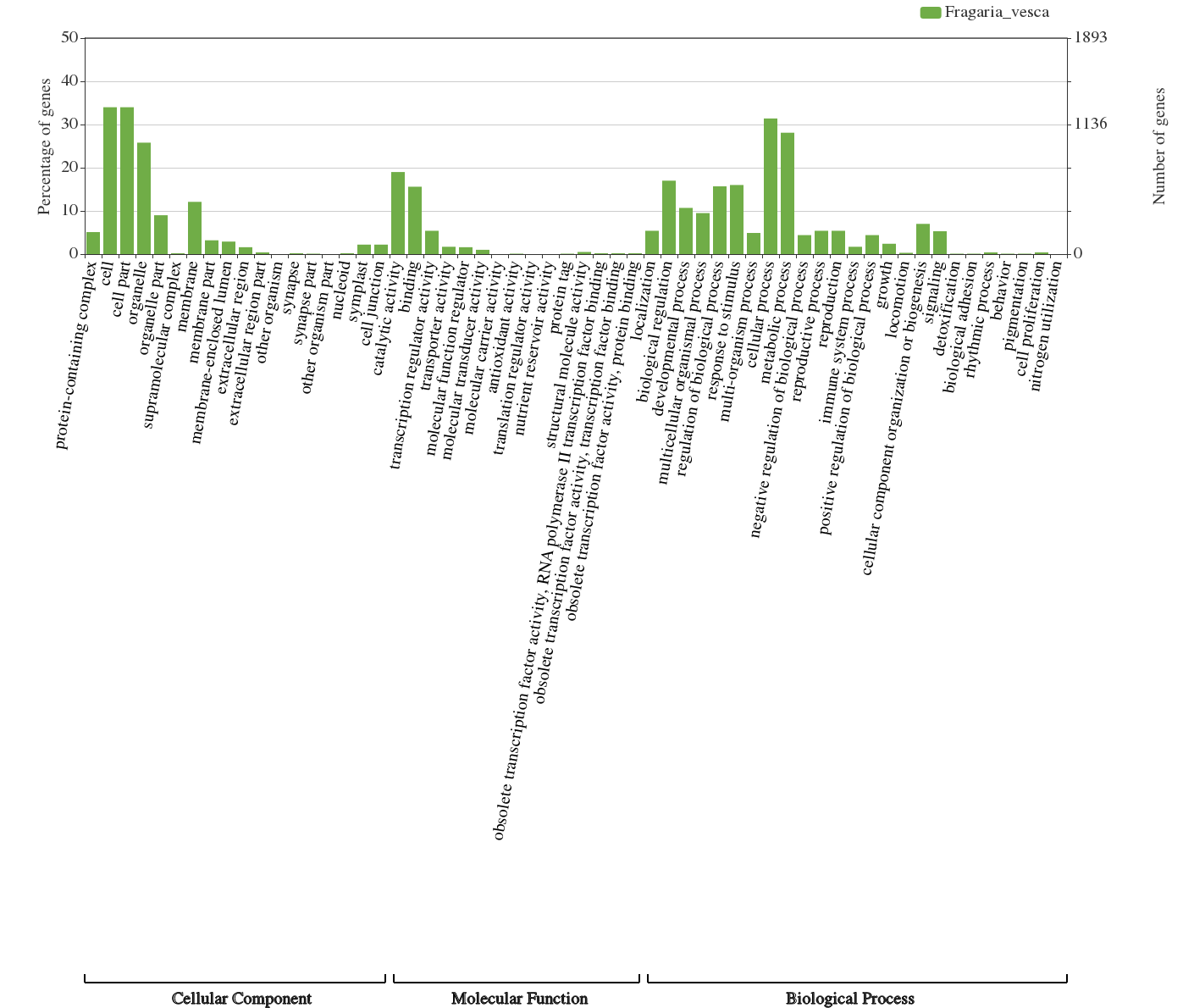


**Supplementary Fig. 13** The statistics of GO classifications in *Fragaria vesca*. The largest proportion of intronless genes were classified as cell and cell part.

**Supplementary Table 1** The predicted isoelectric point and protein molecular of intronless PPR genes in *Pyrus bretschneideri*

| Intronless PPR genes in *Pyrus bretschneideri* | pI | Mw |
| --- | --- | --- |
| LOC103926678 | 6.58 | 71696.39 |
| LOC103926785 | 7.86 | 74845.9 |
| LOC103926956 | 7.42 | 72079.56 |
| LOC103927494 | 5.86 | 11535.57 |
| LOC103927811 | 6.43 | 85392.75 |
| LOC103928913 | 6.46 | 82351.06 |
| LOC103930269 | 9.18 | 36218.15 |
| LOC103930833 | 6.54 | 101712.5 |
| LOC103930984 | 8.97 | 81222.67 |
| LOC103931382 | 9.47 | 56537.21 |
| LOC103931386 | 8.76 | 60810.21 |
| LOC103931545 | 7.91 | 45195.54 |
| LOC103932387 | 8.31 | 68217.64 |
| LOC103932402 | 6.15 | 100629.3 |
| LOC103933370 | 7.59 | 22669.04 |
| LOC103933648 | 6.88 | 70119.83 |
| LOC103933705 | 5.91 | 76466.14 |
| LOC103933811 | 8.46 | 68058.39 |
| LOC103933929 | 5.59 | 74530.26 |
| LOC103934328 | 5.57 | 33891.46 |
| LOC103934401 | 8.35 | 71228.96 |
| LOC103934782 | 6.58 | 71696.39 |
| LOC103935119 | 5.9 | 84790.33 |
| LOC103935671 | 7.07 | 72815.81 |
| LOC103935847 | 8.47 | 75828.61 |
| LOC103937571 | 7.05 | 54996.4 |
| LOC103937703 | 8.17 | 83507.54 |
| LOC103938126 | 6.28 | 107625 |
| LOC103940678 | 9.23 | 45492.98 |
| LOC103942187 | 6.63 | 92302.27 |
| LOC103942975 | 6.16 | 39818.93 |
| LOC103943295 | 8.99 | 53831.35 |
| LOC103944174 | 6.37 | 103566.4 |
| LOC103944275 | 7.87 | 67928.51 |
| LOC103944484 | 7.6 | 56564.26 |
| LOC103944607 | 6.75 | 84767.2 |
| LOC103946076 | 5.62 | 65022.03 |
| LOC103946107 | 8.76 | 54950.87 |
| LOC103946112 | 7.09 | 74830.26 |
| LOC103946306 | 5.65 | 75341.3 |
| LOC103946466 | 8.1 | 96823.94 |
| LOC103946593 | 6.81 | 91311.18 |
| LOC103946944 | 6.42 | 78623.1 |
| LOC103947228 | 6.78 | 60236.75 |
| LOC103947439 | 5.89 | 59443.55 |
| LOC103947845 | 6.23 | 113754.4 |
| LOC103948806 | 6.51 | 21991.58 |
| LOC103948941 | 8.65 | 53522.98 |
| LOC103948942 | 6.76 | 68036.68 |
| LOC103949039 | 6.32 | 103447.3 |
| LOC103949046 | 6.4 | 87336.63 |
| LOC103950767 | 7.88 | 58449.82 |
| LOC103950776 | 7.88 | 58449.82 |
| LOC103950801 | 8.17 | 67615.19 |
| LOC103950802 | 8.17 | 67615.19 |
| LOC103950819 | 7.31 | 66434.5 |
| LOC103950838 | 6.07 | 64507.65 |
| LOC103950877 | 7.27 | 69200.65 |
| LOC103951058 | 7.32 | 80432.82 |
| LOC103951305 | 7.85 | 78611.55 |
| LOC103951694 | 7.31 | 68075.01 |
| LOC103951899 | 6.11 | 54318.66 |
| LOC103952128 | 8.1 | 92814.38 |
| LOC103952948 | 6.87 | 75157.39 |
| LOC103953058 | 9 | 44438.27 |
| LOC103953304 | 6.17 | 59298.36 |
| LOC103953321 | 9.19 | 94801.39 |
| LOC103953451 | 8.4 | 64292.1 |
| LOC103953510 | 5.8 | 56373.83 |
| LOC103954307 | 8.91 | 63484.51 |
| LOC103954369 | 8.25 | 92713.28 |
| LOC103954398 | 7.9 | 52302.56 |
| LOC103954632 | 0 | 0 |
| LOC103955621 | 6.95 | 77107.98 |
| LOC103956160 | 8.47 | 68390.44 |
| LOC103956319 | 6.17 | 85883.94 |
| LOC103956323 | 6.17 | 85883.94 |
| LOC103956453 | 7.23 | 67144.99 |
| LOC103956483 | 7.64 | 32723.59 |
| LOC103956730 | 7.52 | 53030.48 |
| LOC103956872 | 8.59 | 56266.84 |
| LOC103957211 | 8.89 | 87836.16 |
| LOC103957342 | 5.2 | 32019.92 |
| LOC103957495 | 7.97 | 77188.48 |
| LOC103958189 | 6.46 | 75630.62 |
| LOC103958201 | 6.16 | 100168.2 |
| LOC103958209 | 8.34 | 43945.6 |
| LOC103958711 | 7.94 | 66986.28 |
| LOC103959723 | 6.23 | 78555.88 |
| LOC103959901 | 8.6 | 68488.34 |
| LOC103959933 | 5.76 | 58556.36 |
| LOC103960260 | 8.57 | 104162.2 |
| LOC103960506 | 7.9 | 67893.41 |
| LOC103960660 | 6.29 | 93339.12 |
| LOC103960952 | 7.58 | 81098.33 |
| LOC103961018 | 8.61 | 60406.63 |
| LOC103961080 | 7.35 | 69253.04 |
| LOC103961156 | 6.21 | 93485.98 |
| LOC103961272 | 6.46 | 74788.66 |
| LOC103961397 | 6.97 | 100762.4 |
| LOC103962711 | 7.13 | 93483.65 |
| LOC103962796 | 7.52 | 61150.66 |
| LOC103963615 | 6.68 | 77662.53 |
| LOC103963921 | 7.58 | 56243.98 |
| LOC103964189 | 9.18 | 76740.84 |
| LOC103964692 | 8.49 | 61230.33 |
| LOC103964696 | 6.6 | 63547.18 |
| LOC103964938 | 8.78 | 85218.41 |
| LOC103964986 | 6.94 | 80392.37 |
| LOC103965789 | 6.23 | 78514.83 |
| LOC103965860 | 6.88 | 78611.82 |
| LOC103966240 | 8.68 | 68229.87 |
| LOC103966251 | 8.68 | 68343.07 |
| LOC103966284 | 6.61 | 62081.28 |
| LOC103966445 | 7.01 | 67033.76 |
| LOC103966447 | 6.7 | 68086.11 |
| LOC103966820 | 8.58 | 74149.1 |
| LOC103967261 | 6.32 | 79650.11 |
| LOC103967403 | 6.48 | 85509.38 |
| LOC103967413 | 6.52 | 74157.2 |
| Maximum | 9.47 | 113754.4 |
| Minimum | 0 | 0 |
| Average | 7.242583 | 70133.62 |
